# A high-throughput big-data orchestration and processing system for the High Energy Photon Source

**DOI:** 10.1107/S1600577523006951

**Published:** 2023-09-20

**Authors:** Xiang Li, Yi Zhang, Yu Liu, Pengcheng Li, Hao Hu, Liwen Wang, Ping He, Yuhui Dong, Chenglong Zhang

**Affiliations:** a Institute of High Energy Physics, Chinese Academy of Sciences, Beijing 100049, People’s Republic of China; b University of Chinese Academy of Sciences, Beijing 100049, People’s Republic of China; ESRF and Université Grenoble Alpes, France

**Keywords:** beamline software, data acquisition, data management, high-throughput experiment, multimodal experiments

## Abstract

High-data-throughput and multimodal-acquisition experiments will prevail in next-generation synchrotron beamlines. Orchestrating dataflow pipelines connecting the data acquisition, processing, visualization and storage ends are becoming increasingly complex and essential for enhancing beamline performance. *Mamba Data Worker* (*MDW*) has been developed to address the data challenges for the forthcoming High Energy Photon Source (HEPS). It is an important component of the *Mamba* experimental control and data acquisition software ecosystem, which enables fast data acquisition and transmission, dynamic configuration of data processing pipelines, data multiplex in streaming, and customized data and metadata assembly. This paper presents the architecture and development plan of *MDW*, outlines the essential technologies involved, and illustrates its current application at the Beijing Synchrotron Radiation Facility (BSRF).

## Abstract 2

*Mamba Data Worker* (*MDW*) has been developed as a high-throughput, multimodal data orchestration and processing system for the High Energy Photon Source (HEPS). It acts as the main artery system for data and metadata in data acquisition, storage and online analysis. The design and future development plans of *MDW* are discussed.

## 1. Introduction

Synchrotron radiation facilities are essential national infrastructures for cutting-edge scientific research. With advancements in accelerator technology, a large number of fourth-generation synchrotron sources will be built in the next few years across the world, either upgrading from current third-generation sources or built as greenfield facilities. The High Energy Photon Source (HEPS) (Jiao *et al.*, 2018 ▸) is one of the fourth-generation synchrotron facilities with a diffraction-limited storage ring. Many beamlines are under construction in the Phase I project of HEPS, which will be equipped with new-generation optics, sample environments and detectors. The experiment modes at those state-of-the-art beamlines feature high-throughput, multimodal, ultrafast frequency, *in situ* and dynamic experiments, raising significant challenges in experimental control and data acquisition. The *Bluesky*-based data acquisition framework *Mamba* (Liu *et al.*, 2022 ▸) is designed to systematically solve the control and data acquisition tasks for HEPS. After two years of development, *Mamba* has been successfully deployed at several beamlines of the Beijing Synchrotron Radiation Facility (BSRF), a first-generation synchrotron source in China. However, to meet the future big-data challenges, one critical element is still missing in *Mamba* – the orchestration and processing system capable of handling high-throughput and multimodal experiment dataflow. Specifically, *Mamba Data Worker* (*MDW*) has been developed to take over the task within the *Mamba* framework.

The function and performance of *MDW* need to cope with the following trends in data acquisition. Firstly, the wide implementation of next-generation detectors in various kinds of fast experiments (dynamic imaging, X-ray photon correlation spectroscopy, serial tomography, *etc*.) is pushing data throughput to unprecedented levels. For instance, the hard X-ray microscopy beamline (B7) of HEPS is expected to generate an average of 250 TB of data per day. To circumvent the local storage burst that causes data loss, data need to be extracted directly from the detector readout into the storage system. Secondly, the low emittance and high coherence source makes it easier to focus the X-ray beam down to the micrometre and nanometre scales. In fact, nearly two-thirds of the beamlines at HEPS will operate in the microprobe or nanoprobe mode, enabling high spatial resolving power. However, to examine large samples, multi-dimensional scans across real or reciprocal space are required which results in a sharp increase in the number of data points. Furthermore, multi-dimensional scanning experiments are often accompanied by multimodal characterization (Goubran *et al.*, 2019 ▸; Bhargava *et al.*, 2022 ▸). Users aspire to acquire comprehensive information from a single experimental acquisition process to resolve the correlation between structure and functionalities. Therefore, many experiments require simultaneous collection of signals from scattering, diffraction, spectroscopy, microscopy, *etc*. The increase in scanning dimensions, complexity of scan mechanisms and data modality raise tremendous difficulties in data alignment and assembly. A dense topological dataflow network is required to transmit data from multiple data generation to multiple data consuming ends. More importantly, the popularization of *in situ* or dynamic experiments owing to the high beam throughput has pressed the need on real-time data analysis for feedback control, in order to increase the data acquisition efficiency, especially in experiments involving multi-dimensional scan processes and multimodal acquisition. The core mission of *MDW* is hence no longer limited in traditional data acquisition tasks which focus on data collecting and storage but more towards transmitting data into different analysis pipelines at maximum efficiency, and extracting real-time results for decision making and guiding the follow-up experiment process. As the number of scanning dimensions increases, the data processing pipeline becomes longer and computing nodes tend to be more distributed, which is adding further complexity for configuring the processing pipelines. Last but not least, to facilitate online and offline data analysis and promote FAIR (Wilkinson *et al.*, 2016 ▸) data principles at synchrotron facilities, rich metadata and flexible data structure recording mechanisms need to be implemented with the help of *MDW*.

Several software projects have made substantial progress to address the aforementioned data acquisition challenges for third-generation light sources, including *HiDRA* (Fischer *et al.*, 2017 ▸), *ASAPO* (https://asapo.pages.desy.de/asapo/) and *Odin* (Yendell *et al.*, 2017 ▸). As we know, several ongoing projects within the global *Bluesky* (Allan *et al.*, 2019 ▸) community are trying to meet those requirements for future experiments conducted at fourth-generation synchrotron light sources. *MDW* represents HEPS’s attempt to address these challenges and is a critical component of *Mamba* to build a full dataflow lifecycle of advanced light sources, and plays a key role in HEPS’s efforts to accelerate online data acquisition, storage and analysis, facilitating the production of new scientific results. This paper provides an overview of *MDW*’s main functionalities, architecture design, development plan, main technical points and current application status at BSRF.

## 2. Methods

### 2.1. Interfaces with other *Mamba* systems

**Figure 1:**
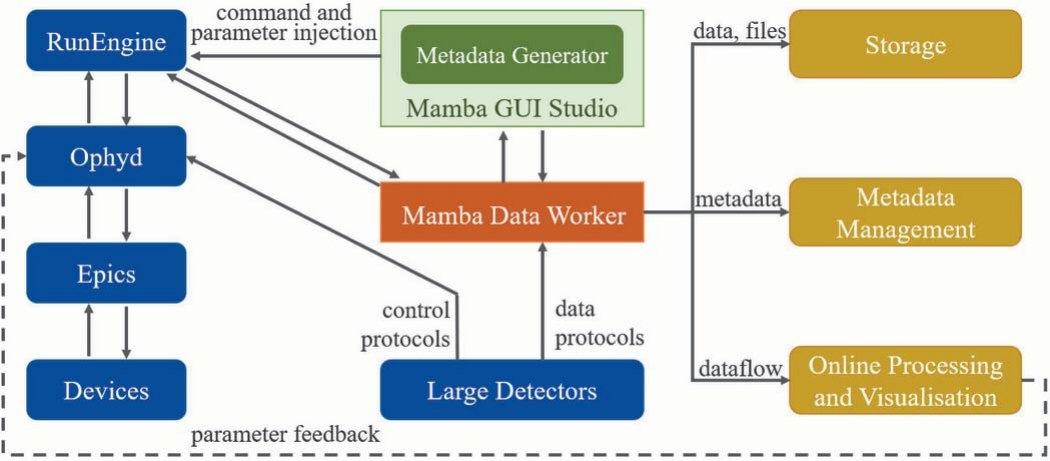
*MDW* interfaces with other systems.

As one of the crucial components of *Mamba*, *MDW* interacts with several other systems, as shown in Fig. 1 ▸. *MDW* receives control signals and data from *Bluesky*’s RunEngine running in the backend of *Mamba*, and retrieves other metadata from *Metadata Generator* (*MDG*) in *Mamba GUI Studio* (*MGS*) where users can input information on experiment description, sample information, *etc*. For low-data-throughput experiments, data from the experiment devices (detectors, motors, *etc*.) are generally sent from *Bluesky* to *MDW* through a subscribe document event (Arkilic *et al.*, 2015 ▸). For experiments involving high-data-throughput detectors, *MDW* directly fetches data from the detectors and transmits data to the downstream ends which include storage, *Mamba* frontend, analysis module, scientific metadata database, *etc*. *MDW* can either be informed of the starting of a new acquisition by the *Mamba* frontend, *Bluesky* RunEngine or data streams of the detector.

### 2.2. Framework design of *MDW*

**Figure 2:**
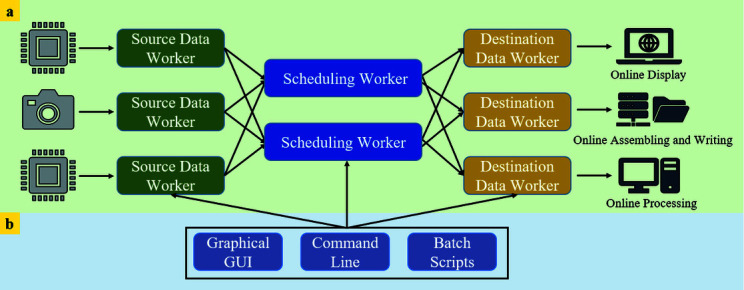
Components of *MDW*, showing (*a*) the data plane and (*b*) the control plane.

The communication entity defined by *MDW* is called the worker. The worker is composed of three distinct components, as illustrated in Fig. 2 ▸: source data worker, scheduling worker and destination data worker. The acquisition, streaming and online processing of experimental data are split into two separate and major parts referred to as the control plane and data plane. By separating the control plane and data plane, it is possible to execute them on different devices, which in turn enhances the system’s flexibility and will enable rapid processing with dedicated hardware in the future. In the control plane, the configuration of workers in the data plane can be performed through a graphical user interface (GUI), command line interface and other methods. In the data plane, ZeroMQ serves as the communication protocol between workers, and all data are currently sent over the ZeroMQ socket.

The source data worker is responsible for collecting data and metadata of different formats [Tiff, HDF5 (Folk *et al.*, 2011 ▸), raw data stream] from measurement devices in various scanning modes. Subsequently, the source data worker forwards the data stream to the scheduling worker via ZeroMQ in real time. The scheduling worker is responsible for scheduling the experimental stream to multiple destination data workers who require the data stream according to the *MDW* application programming interface (API) or configuration file. The data stream is ingested by destination data workers which cater to diverse application requirements in synchrotron radiation. Such requirements include real-time data visualization facilitated by GUIs and various frameworks, online data assembling and writing for multiple data streams according to specified HDF5 data structure defined by beamline stations, and online data processing tasks. The destination worker exhibits inherent functionality owing to its hardcoded implementation, enabling it to discern the necessary actions to be taken. Each individual destination worker assumes the responsibility of determining the specific operations to be carried out on the received data. Furthermore, it is possible to dynamically add a destination on-the-fly in real time during the acquisition process. Users should have the capability to transmit a configuration message to *MDW* through the *MDW* API, thereby enabling the scheduling worker to add a route to the newly designated destination. Subsequently, the newly added destination worker becomes capable of receiving data.

**Figure 3:**
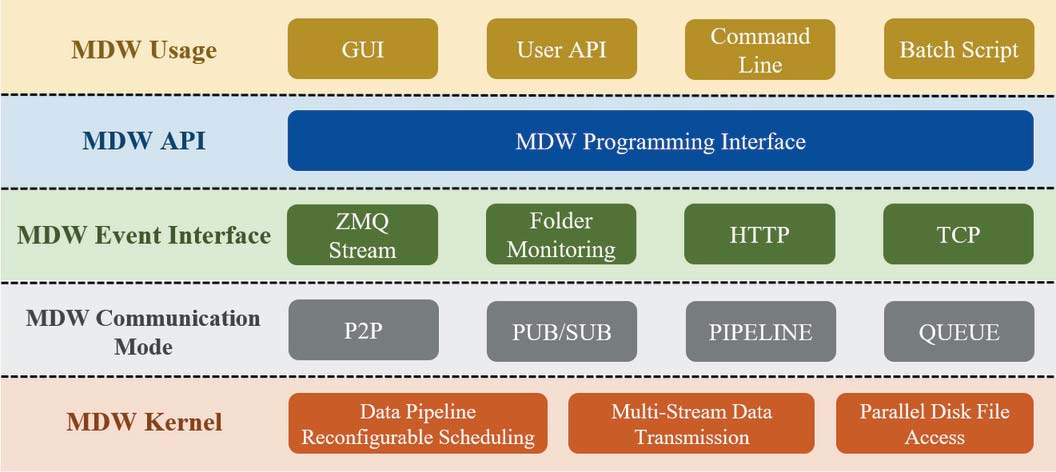
*MDW* hierarchy.

The *MDW* framework hierarchy is shown in Fig. 3 ▸ and consists of five layers from the bottom up: the internal kernel layer, communication pattern layer, event interface layer, programming interface and usage methods.

(i) The kernel layer of *MDW* consists of three modules: dynamic reconfigurable data pipeline scheduling, high-throughput multi-stream data parallel transmission and high-performance parallel disk file access. These modules aim to solve the complex dataflow scheduling problem, enhance network transmission performance and disk file access performance.

(ii) *MDW* manages dataflow based on ZeroMQ. The communication pattern layer includes various communication patterns, such as P2P, publish–subscribe (PUB–SUB), PIPELINE processing and QUEUE. The various communication patterns will support diverse data-consuming mechanisms in the downstream data application ends.

(iii) The event interface in source data worker supports multiple protocols to acquire different types of data, such as detector API, ZeroMQ streams, temporary files and HTTP streams. It enables simultaneous acquisition of multiple modal types of data.

(iv) *MDW* provides programming interfaces for programming and extension, including worker configuration interface, data link establishment interface and experiment processing sequence configuration interface, among others.

(v) Users can achieve customized experimental dataflow scheduling and processing through the *MDW* programming interface wrapped with graphical GUI, user API, command line interface, batch scripts and other common usage methods in the control plane. This facilitates meeting different needs of beamline stations.

**Figure 4:**
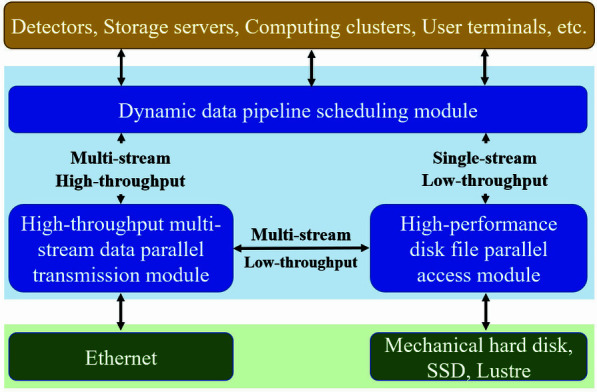
Relationship between the three kernel modules of *MDW*.

Figure 4 ▸ illustrates the intrinsic logical relationship between the various research subjects of *MDW*. The dynamic reconfigurable data pipeline scheduling module provides a unified view of the experimental data pipeline between light source systems such as detectors, storage servers, computing clusters and user terminals, and provides flexible distribution and scheduling of the experimental dataflow pipeline. Based on the establishment of the experimental data pipeline, the high-throughput multi-stream data parallel transmission module and the high-performance disk file parallel access module improve the performance of experimental data transmission and storage.

### 2.3. Dynamic data pipeline scheduling

To meet the diverse and developing experiment data pipelines at different beamlines, *MDW* provides a dynamic data pipeline creation and scheduling module. The module aims to abstract the common features of different data pipelines by abstracting the entities responsible for data distribution and scheduling, including measurement devices and experimental phases, *etc*. The specific data allocation and scheduling mechanism of each entity is then hidden behind the abstraction layer which enables rapid and flexible data pipeline creation at different application scenarios. Dynamical data pipelines scheduling is essential for the user to interact and control experiment processes. This module listens to control commands from other workers or user programming interfaces. It then parses these control commands and dynamically changes the topological path between the data source and destination as required by the command.

To allow an easier and more streamlined experience for configuring data pipelines, a visual workflow engine based on *Orange* (Demšar *et al.*, 2013 ▸) is currently being developed as the frontend for *MDW*. Users can quickly build data flow diagrams between various types of workers by dragging and dropping widgets on the *Orange* canvas. Each widget is a self-contained worker, encapsulating interface display, interaction control and some data processing functionalities. This will particularly benefit the orchestration of data pipelines for multimodal experiments.

### 2.4. Multi-stream data transmission

The trend of high-throughput and multimodal data acquisition requires improvements on data transmission throughput to enable the transfer of multimodal data types from source data workers to multiple receivers in the form of streams. *MDW* uses standard Python libraries such as JSON to implement serialization. The data from different detectors are transmitted using independent data streams to improve network bandwidth utilization. In multimodal experiments, it is common that data volume and structure vary among each modality; therefore, the bandwidth and computing complexity with respect to each data pipeline will vary significantly, the asynchronous data streams need to be synchronized with the metadata, and the raw and processed data ultimately need to be correlated with each other; thus, developing a reliable data alignment scheme is essential.

For low- and medium-data-throughput experiments, if the data are collected through *Bluesky*, which already has its own data alignment mechanism, *MDW* directly subscribes to *Bluesky* events to obtain data and metadata which are subsequently sent to a receiver. This can be done through either polling or pushing mechanisms, depending on the specific use case. For instance, in a step scan, the destination data worker may poll the data for each step and receive the data point-by-point. However, for fly scans that are currently not processed through *Bluesky* but rather are stored as separate HDF5 files by PandABox (Li *et al.*, 2023 ▸) and Xpress3 (Crawford *et al.*, 2018 ▸), *MDW* possesses the capability to append pertinent fields, such as the acquisition number of each data frame, through the source data worker which can further facilitate data synchronization. This enables *MDW* to efficiently read and sequentially bundle a batch of data into blocks in real time, whose size can be configured through a configuration file. For high-data-throughput experiments involving large area detectors, *MDW* has to fetch raw data streams directly from the detectors. Essential metadata information, such as timestamp or index, which come with the data streams, will be used for aligning the data streams in the subsequent pipeline.

### 2.5. Parallel disk file access

Efficient file access is essential for high-performance data storage and retrieval. This requires the implementation of several key functionalities, including online compression, multi-level file assembly and parallel writing of multimodal data. *MDW* provides conventional compression methods such as Gzip (Shah & Sethi, 2019 ▸), Blosc (Alted, 2010 ▸) and LZ4 (Bartík *et al.*, 2015 ▸), and also plans to support advanced compression modes based on artificial intelligence. Data assembly in *MDW* is flexible and customizable, allowing users to define their own data structures at different beamlines. Generally, for experiments with low and medium data volume, all data are a single Nexus (Könnecke *et al.*, 2015 ▸) file; whereas, for experiments with large data volume, *MDW* supports writing data simultaneously with desired structure into multiple HDF5 files which are linked to a main NeXus file. *MDW* supports reading and writing of multiple file formats, such as Tiff, HDF5 and Nexus. To support simultaneous read access from multiple processes, *MDW* will incorporate the single-writer/multiple-reader (SWMR) and virtual dataset (VDS) techniques developed by the HDF Group (Rees *et al.*, 2015 ▸).

### 2.6. Metadata parameter configuration

**Figure 5:**
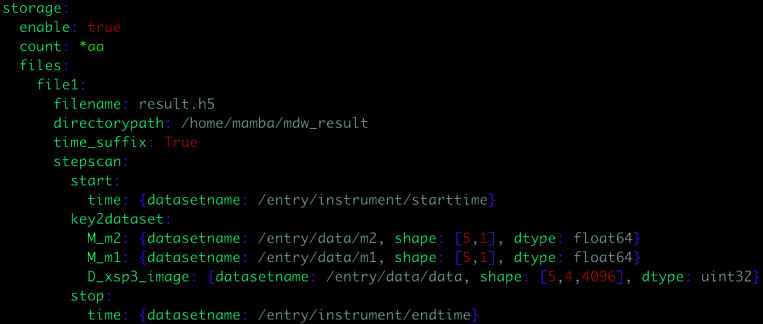
Flexible configuration to write certain parameters of the metadata in a custom data structure as HDF5.

Experimental processes involve not only raw data but also rich metadata such as environment parameter, sample, user information and more. There are several mechanisms for associating data and metadata with each other. The main experimental data and metadata associated with the plan is through *Bluesky*’s pre-assembled document. Metadata can also be obtained from *MDG* or other user input information to which designated paths will be assigned, and then the path-containing metadata are sent to *MDW*, which associates the data and metadata based on the path. To enhance the parametric configuration capability of *MDW*, each beamline station can customize the metadata format, port address and other parameters according to their own requirements. This avoids redundant development efforts and improves development efficiency. To facilitate configuration, we have designed two common methods specifically for the *MDW* system: the YAML-based parameter configuration function and the frontend GUI. Figure 5 ▸ demonstrates the YAML-based configuration file that simplifies the configuration of data and metadata, such as *MGS*-related parameters, user login authentication and detector acquisition, through an easy-to-understand syntax.

## 3. Results

**Figure 6:**
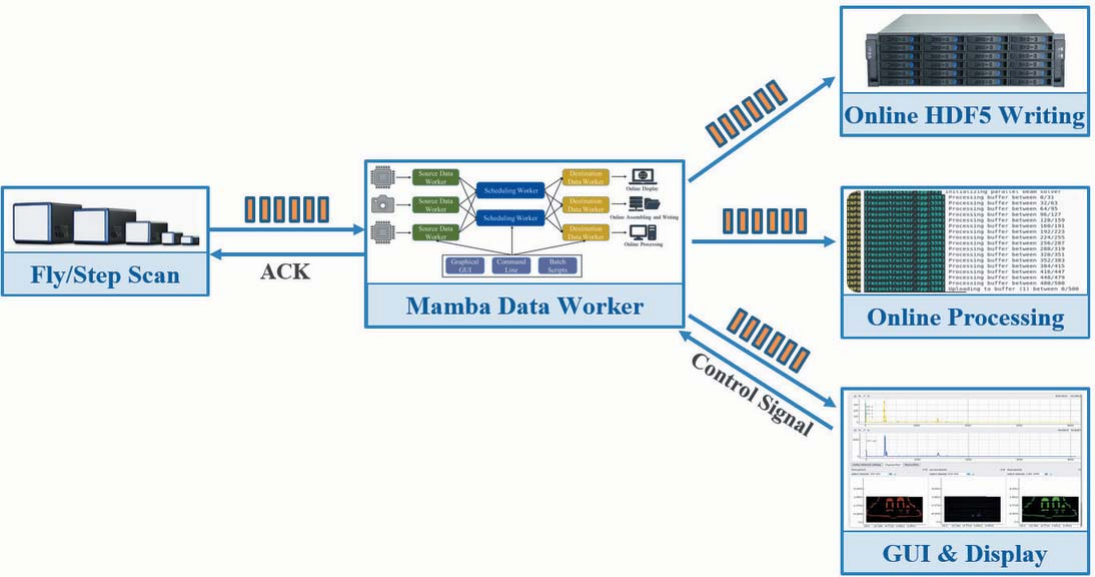
*MDW* deployment in beamline stations.

So far, some architecture of *MDW* has already been developed and some of its functionality has been validated along with the successful implementation of *Mamba* prototype software at BSRF. Although the data throughputs are not exceedingly high for the current experiments, *MDW* has demonstrated strong versatility and flexibility to support experiments at different beamlines, methodologies, measurement devices and scan mechanisms, as shown in Fig. 6 ▸.

One application scenario for *MDW* is the tomography experiment at the 3W1 beamline of BSRF, where data generated from the in-house-developed imaging detector (Dhyana 6060-CP FSI version, pixel array 6K × 6K, maximum frame rate up to 19 Hz) need to be highly multiplexed. By developing an interface with the scan mechanism module of *Mamba*, *MDW* can assemble the data from multiple scans and commands (acquiring tomography datasets, flat and dark images *etc*. in a batch) into one master file and multiple sub-data files which adds great convenience for data management and analysis. *MDW* allows online transformation of data structure and easy user configuration from the GUI to facilitate visualization and analysis process. The data stream is directly injected into *RECAST3D* (Buurlage *et al.*, 2018 ▸) for real-time tomography reconstruction, visualization and segmentation. Another application of *MDW* is the on-the-fly X-ray fluorescence mapping experiments at the 4W1B beamline of BSRF. *MDW* managed to fetch the raw data generated in the PandABox and Xpress3 devices and assemble them into an HDF5 file in real time during a multi-dimensional continuous scanning experiment. To provide a user-friendly experience that allows users to acquire and process data in one-click, *MDW* managed to interface directly with *PyMCA* (Solé *et al.*, 2007 ▸) analysis packages and forward the real-time elemental distribution results to the *Mamba* GUI for visualization and further scanning region of interest extraction.

## 4. Conclusion

*MDW* is a critical component of the scientific software system for HEPS, serving as the main artery for circulation of data in data acquisition, online analysis and storage. Its primary purpose is to establish a topological dataflow pipeline that draws upon multiple generation and application ends, automatically capturing metadata and raw data throughout the experiment, while accomplishing dataflow processing tasks such as compression, sampling, assembly and denoising. *MDW* enables efficient data multiplexing, resolving the complexities associated with asynchronous online data processing for high-throughput and multimodal experiments, thereby enhancing experimental efficiency. After two years of development, the basic framework of *MDW* is nearing completion. *MDW* has been demonstrated to be robust and reliable to support various experimental methods across multiple BSRF beamlines. However, the real high-throughput and multimodal data acquisition scenario is yet to come. To address the data challenges at HEPS in the future, the software architecture and the modular performance of *MDW* will continue to be refined.
